# Bacteriophages and food safety: An updated overview

**DOI:** 10.1002/fsn3.3360

**Published:** 2023-05-04

**Authors:** Ali Imran, Umber Shehzadi, Fakhar Islam, Muhammad Afzaal, Rehman Ali, Yuosra Amer Ali, Anamika Chauhan, Sunanda Biswas, Sadaf Khurshid, Ifrah Usman, Ghulam Hussain, Syeda Mahvish Zahra, Mohd Asif Shah, Adil Rasool

**Affiliations:** ^1^ Department of Food Sciences Government College University Faisalabad Pakistan; ^2^ Department of Clinical Nutrition NUR International University Lahore Pakistan; ^3^ Department of Food Sciences, College of Agriculture and Forestry University of Mosul Mosul Iraq; ^4^ Department of Home Science Chaman Lal Mahavidyalaya Landhora Haridwar India; ^5^ Sri Dev Suman University Tehri India; ^6^ Department of Food & Nutrition Acharya Prafulla Chandra College Kolkata India; ^7^ Neurochemicalbiology and Genetics Laboratory (NGL), Department of Physiology, Faculty of Life Sciences Government College University Faisalabad Pakistan; ^8^ Department of Environmental Design, Health and Nutritional Sciences Allama Iqbal Open University Islamabad Pakistan; ^9^ Institute of Food Science and Nutrition University of Sargodha Sargodha Pakistan; ^10^ Adjunct Faculty University Center for Research & Development, Chandigarh University Mohali India; ^11^ Department of Management Bakhtar University Kabul Afghanistan

**Keywords:** bacteriophages, food safety, foodborne illness, phages

## Abstract

Despite significant advances in pathogen survival and food cleaning measures, foodborne diseases continue to be the main reason for hospitalization or other fatality globally. Conventional antibacterial techniques including pasteurization, pressurized preparation, radioactivity, as well as synthetic antiseptics could indeed decrease bacterial activity in nutrition to variable levels, despite their serious downsides like an elevated upfront outlay, the possibility of accessing malfunctions due to one corrosiveness, as well as an adverse effect upon those the foodstuffs' organoleptic properties and maybe their nutritional significance. Greatest significantly, these cleansing methods eliminate all contaminants, including numerous (often beneficial) bacteria found naturally in food. A huge amount of scientific publication that discussed the application of virus bioremediation to treat a multitude of pathogenic bacteria in meals spanning between prepared raw food to fresh fruit and vegetables although since initial idea through using retroviruses on meals. Furthermore, the quantity of widely viable bacteriophage‐containing medicines licensed for use in health and safety purposes has continuously expanded. Bacteriophage bio‐control, a leafy and ordinary technique that employs lytic bacteriophages extracted from the atmosphere to selectively target pathogenic bacteria and remove meaningfully decrease their stages meals, is one potential remedy that solves some of these difficulties. It has been suggested that applying bacteriophages to food is a unique method for avoiding bacterial development in vegetables. Because of their selectivity, security, stability, and use, bacteriophages are desirable. Phages have been utilized in post‐harvest activities, either alone or in combination with antimicrobial drugs, since they are effective, strain‐specific, informal to split and manipulate. In this review to ensure food safety, it may be viable to use retroviruses as a spontaneous treatment in the thread pollution of fresh picked fruits and vegetables, dairy, and convenience foods.

## Graphical abstract

Bacteriophage bio‐control, a leafy and ordinary technique that employs lytic bacteriophages extracted from the atmosphere to selectively target pathogenic bacteria and remove meaningfully decrease their stages meals, is one potential remedy that solves some of these difficulties. It has been suggested that applying bacteriophages to food is a unique method for avoiding bacterial development on vegetables.

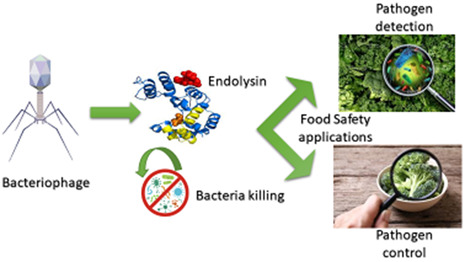

## 1 INTRODUCTION

Dieticians and other health specialists all over the world highly advocate eating fresh fruit and vegetables because they are high in vitamins, minerals, and other nutrients (Fan et al., 2009). When compared to additional nutrition categories such as meat, fish, and dairy, fresh produce continues to be a major reason for epidemics of contaminated food sources. Since 1990, there have been over 400 emergences of foodborne infection related to produce. Fresh meals like tomato, leafy greens, other fruit and vegetables, as well as sprouted seeds like clover, mung beans, and alfalfa, are the most commonly connected with outbreaks (Murray et al., 2017). Meanwhile, better fruit is often grown on open fields, and gathered product is more prone to diarrheal disease from staff, pollen, water sources, thread liquid, predators, feces, and many other factors (Fan et al., 2009). Between 2010 and 2018, the Centers for Disease Control (CDC) recorded 51,710 response of foodborne illness. Consuming contaminated food has been linked to 25,388 outbreaks, 12,055 of which needed hospitalization, and 950 of which resulted in fatal illnesses (Jagannathan et al., 2022). Besides causing foodborne illnesses, microorganisms may reduce the quality of vegetables, resulting in spoilage. Since fruits and vegetables include living tissue, which makes them extremely perishable and prone to loss, they must be kept alive throughout the production process and up until sale. Food waste from farm to fork has a significant economic and environmental impact (Rawat, 2015). Post‐harvest losses of fruits and vegetables can range from 30% to 40% globally, and they can be significantly higher in underdeveloped countries. Furthermore, it is anticipated that 20% of product in the US spoils each year (Barth et al., 2009). It is essential to take into account strategies for boosting productivity and decreasing waste. The need for non‐chemical methods of food protection is still there due to rising organic food production and rising health consciousness. Over time, a diversity of tactics takes suggested stop contamination of fresh fruit and vegetables. In order to maintain safety via bio‐sanitization and bio‐preservation, utilized the bacteriophages as bio regulator agents takes remained researched then put into practice which diet business. Bacteriophages, sometimes referred to as phages, are bacterial worms that may reproduce inside their bacterial hosts, lysing their cells and finally killing them. The name “bacteriophage” was initially adopted by microbiologist Felix d'Herelle after discovering this unidentified virus in samples of human feces. In order to mature and reproduce, bacteriophages are obligate intracellular parasites that require living hosts. They are among the creatures that are most common. Phages may be found anywhere; however, they tend to be more common in settings with the suitable host microbes. A number of phages that may infect starter cultures and inhibit their growth provide a constant threat to some food firms, particularly those in the dairy and fermentation industries (O'Sullivan et al., 2019).

Instead, different phages are used to stop food from rotting and from being infected with harmful bacteria, hence reducing food waste and the risk of catching foodborne illnesses. It is well acknowledged that a variety of food products post a threat to humanoid health because of pervasive microbial adulteration, which can result in disease or death (Ahmad, 2023; Usman et al., 2023). Products, fisheries, milk products, chicken, and veggies are examples of items that are routinely commercially manufactured through semi husbandry, large founder, or inter shipping. There is a greater chance of contamination as a result. It has been shown that phages may be effectively utilized to lower microbial pollution on food products, hence enhancing food safety Antimicrobials are commonly present in a variety of products, including unpasteurized milk, poultry, cheeses, vegetables and fresh fruits (Greer, 2005; Islam et al., 2022). Numerous research has been conducted to assess how bacteriophages may be used to create antibacterial agents to enhance microbiological food safety as well as their ability to reduce pathogen and spoilage microorganisms. This review to ensure food safety, it may be viable to use retroviruses as a spontaneous treatment in the thread pollution of fresh picked fruits and vegetables, dairy, and convenience foods.

## 2 FOODBORNE ILLNESS LINKED TO FOOD

Contamination is one of the leading contributors of disease and death around the globe. Roughly 250 gastrointestinal diseases were identified so far, and around 9.4 million instances of foodborne outbreaks are registered in the U.S. every year, resulting in about 56,000 hospitalizations with 1300 fatalities. The majority of these cases are caused by a specific group of food‐borne pathogens such as *Shigella*, *Salmonella*, *Campylobacter*, *Listeria monocytogenes*, and *Escherichia coli pathotypes* other enteric microorganisms (Murray et al., 2017; Scallan et al., 2011). Because of the strong demand for fresh produce, the majority of these occurrences are linked to insufficient thermal stowage, besides microbiologic dirtied gear, but ingesting food after hazardous causes is continuously implicated (Żaczek et al., 2015). Bacteriophages are specialized viruses that attack bacteria as an alternative to antibiotics by rupturing the cell wall to address the emergence of bacterial resistance (Bragg et al., 2014). Bacteriophages with either RNA or DNA genomes can produce endolysin enzymes, which cleave peptidoglycan to lyse cell walls. Moreover, the genome of bacteriophages contains proteins known as amurins, which prevent the production of cell walls and cause cell walls to rupture (Woznica et al., 2015).

## 3 PHAGE RESEARCH TO IMPROVE FOOD SAFETY

Since discovery of bacteriophage by Francis Type of circuit and Walter d'Herelle a century earlier, researchers have shown that phages can cure microbial enterococcus illnesses like cholera, correctly selected, and giardiasis in addition to a diversity of acute or prolonged pathogens in fields like cardiology, gastroenterology, cardiology, neonatology, and multiple surgeries (Wittebole et al., 2014). Since then, such infectious beings have been utilized for a variety of farming applications, as well as for animal and human uses, even though to our understanding, advertising bacteriophage therapy on local food have never been recorded (Sillankorva et al., 2012). Fresh produce‐related episodes of contaminated food have highlighted the importance of practical methods to eliminate harmful bacteria from food. It has been demonstrated that traditional commercial sanitizers fail to eliminate harmful bacteria from either the skins of fruits and vegetables (Bhardwaj et al., 2015).

Radioactivity, consumable covering, nitrogen oxides, ultraviolet, climate‐controlled storing, potassium permanganate, water, and sometimes perhaps viral proteins are just a few of the techniques that were researched to discover more efficient options to assure bacterial elimination on healthy produce (Mahajan et al., 2014; Islam et al., 2022). A few of the methods that have been studied to find more effective ways to ensure bacterial elimination on healthy produce include radioactivity, consumable covering, nitrogen oxides, ultraviolet, climate‐controlled storage, potassium permanganate, water, and occasionally perhaps viral proteins. Microbes are effective and affordable options for organic management since they do not degrade the flavor of new food the way conventional cleaning methods can. The use of viral formulations was already investigated for assess overall bio‐control capability of bacteriophage toward the few dietary pathogens linked to sickness epidemics of veggies and fruits. The unexpected character of the outcomes, however, is one of the problems impeding the use of bacteriophage for phytoremediation there in the local food sector. Nevertheless, it is currently thought because insufficient treatment in during production of the viral concentration and a lack of knowledge of bacteriophage ecology were the key reasons for the error (McCallin et al., 2013).

## 4 BACTERIOPHAGES—EVOLUTION AS ANTIMICROBIALS

Bacteriophages dominate bacterial cells in the environment and in the intestines of both visceral and humanoid types by a factor of 10. They make up the vast bulk of the planet's species. Phage genome sizes range from 3.4 kilobases (kb) to around 500 kilobases (kb), and each phage genome contains multiple genes and proteins that have not yet been fully characterized (Romero‐Calle et al., 2019). The two distinct life cycles that phages go through are lysogenic and lytic. Lysogenic phages inject their viral genome into the host's genetic material, whereas lytic phages kill and destroy infected host cells (Garvey, 2020). Phages with a restricted host range and improved selectivity utilized a variety of host receptors, such as proteins, sugars, and lipopolysaccharides, to bind to the host cell (Batinovic et al., 2019). When its prophase's gene is inserted by lytic bacteriophage, or chromosome, of the host bacterium, several prophages (poly‐lysogenic strains) may be present (Marcó & Mercanti, 2021). Lysogenic phages are important for the biosphere because they regulate population size, promote biomass turnover, and release nutrients in places anywhere lysogenic species might modify in microbial communities (Braga et al., 2020).

Prior to the development of antibiotics, the antibacterial activity of phages was long regarded for the treatment of infectious diseases. The notion of employing microbes was initially supported by the microbiologists Felix d'Herelle, and phages were formally identified in 1917. D'Herelle acknowledged the phages' biocompatibility with the host patient while maintaining their selectivity and potency in eliminating dangerous germ cells. After providing mental healthy hens with *Salmonella gallinarum*, D'Herelle undertook many studies employing viruses and bacteria for intravascular (IV) therapy versus invading microbial illnesses. The first time that phages that might infect *Shigella dysenteriae* were used to treat bacillary dysentery was in 1921 (Braga et al., 2020). In India, phage therapy controlled near a significant drip in transience (62.8%–8.1%), and phages targeted at the cholera bacteria were also added to village water supplies to stop outbreaks. Phage therapy was exceeded by the discovery and development of antibiotics in the 1930s (sylph medicines) and 1940s (penicillin). Viral investigation has problems with unreliable findings, administration dosages, repeatability challenges, and a lack of genetic information. It is also necessary to consider difficulties such as huge composition, production, durability, including preservation (Garvey, 2020). Poland maintained its bacteriophage investigation, which has proven effective combating AMR diseases, although the United States and Continental Europe abandoned the concept. When injected with ipratropium, bacteriophage R was shown to be less efficient versus K1 *E*. *coli* versus 8 dosages of the drug doxycycline (IM). If pathogenic organisms were not discovered in the muscular, heart, plasma, or hepatic of medicated animals 16 hours after treatment, phage clearance was obvious. Research done in Eastern Europe has shown that phages given parenterally or entirely have been effective for around 90 years without endangering the health of the patients (Tang et al., 2019). Phage treatment may be able to provide much‐needed alternatives when present therapeutic methods are failing, given the obvious and worrisome threat that AMR poses. The discovery of bacteriophage treatment might give methods are usually throughout the event of *Clostridioides difficile* infection (CDI), an MDR microbial sickness with a significant fatality rates. In the United States, the United Kingdom, the Republic of Poland, Belgium, and Georgia there are presently six medical centres that provide bacteriophage for the management of contagious disorders (Selle et al., 2020).

## 5 BACTERIOPHAGES FOR BIOCONTROL OF PATHOGENS IN FOOD

**FIGURE 1:**
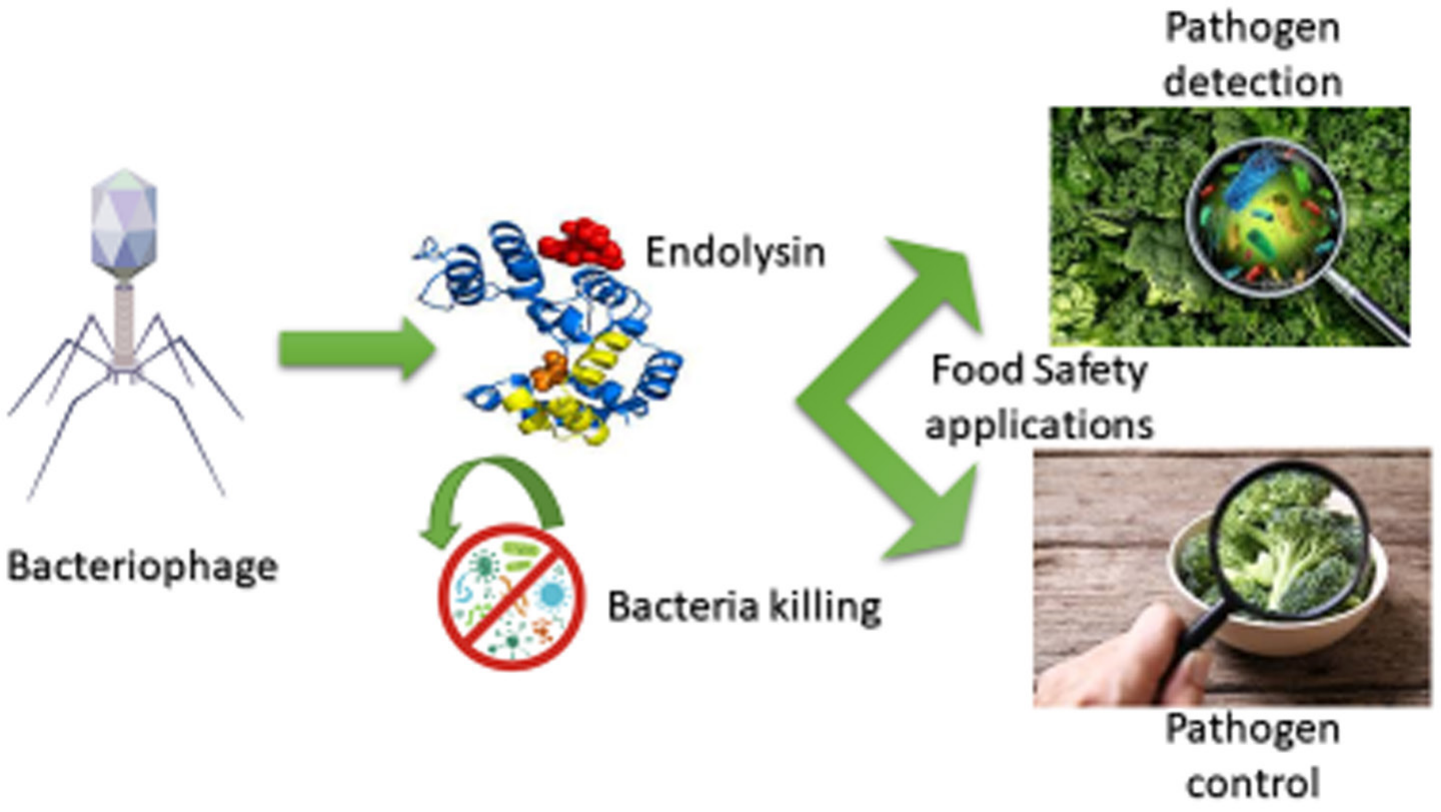
Bateriophage endolysin for food safety application.

Microbe pesticide assays get the ability to significantly enhance bacterial quality because to their long tradition of healthy use, comparatively straightforward processing, and powerful and targeted antibacterial, as indicated in Table 1 and Figure 1.

**TABLE 1:** Application of bacteriophages in food safety and reported outcomes.

Pathogens	Reported results	References
*Listeria monocytogenes*	Elimination of cut melon and apples. combination with nisin. At 4°C, there is no combination phage‐nisin activity in beef. elimination from soft cheese with a surface‐ripened red stain	Garcia et al. (2008)
*Salmonella* spp.	Salmonella counts on melon slices dropped by ~3.5 logs at 5 and 10°C and ~2.5 logs at 20°C following the application of a four‐phage cocktail, but there was no bacterial reduction on apple slices after phage administration	Whichard et al. (2003)
*Escherichia coli*	Decrease in drinkable water and rectal administration (cattle)	Raya et al. (2006)
*Shigella sonnei*	A five‐phage, *Shigella* specific cocktail was administered to a variety of RTE meals, such as smoked salmon, lettuce, melon, corned beef, and pre‐cooked chicken, which decreased the recovery of *Shigella* ~ 1.0–1.4 logs when matched to control	Soffer et al. (2017)
*Campylobacter*	Reduced cecal concentration counts (broilers)	Wagenaar et al. (2005)

### 5.1 Bacteriophage to control *Listeria monocytogenes* contamination

The identification and eradication of *L*. *monocytogenes* is crucial to guaranteeing the chain's safety, particularly in RTE meals, since it may live and develop at the normal low level (2–8°C) employed numerous foods throughout their storage and distribution. Accordingly, several studies have demonstrated that using bacteriophages on a variety of foods, notably RTE meals, is beneficial for lowering *L*. *monocytogenes* infection. As contrasted to vanillin and salt lactose there at ambient usage level of 6–8°C, a commercialized monophase preparations (i.e., phage preparedness comprising about one solitary bacteriophage) against *Listeria* was demonstrated to be higher successful in reducing the concentrations of *L*. *monocytogenes* in sliced ham (Selle et al., 2020). The same monophage preparation was demonstrated to be efficient in reducing *L*. *monocytogenes* on the superficial of various deli meats. Roast beef and turkey slices were frozen at 4 and 10°C, respectively. The *Listeria* specific phage not only acted as a stand‐alone *L*. *monocytogenes* inhibitor but also improved the efficiency of other antimicrobials when coupled with potassium lactate or sodium diacetate (Chibeu et al., 2013).

All of these research utilized the same bacteriophage preparation. A realistic way to include a larger variety of mark species also reduce the possibility that resilient microorganisms would arise is to combine numerous bacteriophages to produce a “phage cocktail.” In certain goods that had been intentionally dirtied through *L*. *monocytogenes*, such as lettuce, cured salmon, Gali apples pieces, and hard pasteurized cheese, the levels of the pathogen decreased by 0.7–1.1 logs when the technique was applied. The effect of the *L*. *monocytogenes*‐specific cocktail on pre‐made, freezer meals was investigated in the same experiment. Cycles of freezing and thawing, phage cocktail treatment, and an experimental *L*. *monocytogenes* infection were all applied to the meals. The findings revealed a 2.2 log decrease in *L*. *monocytogenes*, indicating possessing of bacteriophage might be a useful strategy for reducing *L*. *monocytogenes* in food below “storage abuse” circumstances, such as frequently defrosted throughout stowage, either intentionally or unintentionally (Perera et al., 2015).

### 5.2 Bacteriophage to control *Salmonella* contamination

The number of *Salmonella* strains that are susceptible on overall exteriors examined by 2–4 logs was significantly reduced by a cocktail of *Salmonella* specific bacteriophages, according to recent research, but it was unable to reduce the number of an additional strain of *Salmonella* (*Salmonella Paratyphi* B S661) that was resistant to the cocktail of phages in vitro. Whenever the bacteriophage mixture was altered to include monoclonal antibodies addressing this mutated version, the revised formulation demonstrated a significant drop (2 logs) of *S*. *paratyphi* B S661 from the surfaces whilst remaining efficient it versus formerly definable components (Woolston et al., 2013). This study shows that bacteriophage mixtures may be simply changed to address specific microbes, such as those that are widespread in specific food processing plants or those that have troublesome mutations that make them resistant to phage. *Salmonella* may be eliminated from food and surfaces used in food preparation by combining bacteriophages. For instance, the *Salmonella* specific cocktail reduced the *Salmonella* levels in chicken sections that were experimentally infected when treated alone. This effect was increased when the bacteriophage was mixed with standard chemical sanitizers. The bacteriophage cocktail significantly decreased Salmonella populations when sprayed to the exterior of chicken breast steaks or when the fillets were dipped into a vessel holding the virus solution (Sukumaran et al., 2015).

Moreover, whether the fish were kept either anaerobic or changed weather systems, this bacteriophage combo greatly decreased the amount of *Salmonella*. Because food producers frequently change ventilation parameters to reduce microbial activity and extend consumer expiration lives, that last statement may have actual ramifications. Researchers discovered that a specific bacteriophage, SJ2, greatly decreased the level of *Salmonella* in fluid ova or swine belly, with the decrease being stronger at lower degrees. There was little distinction in the proportion of resilient replicas across microbe or unprocessed chuck roast collections, yet here are significantly higher resistance replicas inside the microbe eggs sample preparation. The authors then looked for resistance in the *Salmonella* colonies that had survived. The researchers hypothesized that the variance in the frequency of resilient *Salmonella* separates may have been caused by variations in the microbiomes of the two diets and the nourishment medium (solid vs. liquid; Hong et al., 2016).

### 5.3 Bacteriophage to control *E*. *coli* contamination

According to recent investigations, fresh vegetables, fresh milk polluted with *E*. *coli*, and (UHT) preserved milk have all been effectively treated utilizing *E*. *coli*‐specific phage formulations. One phage dramatically abridged amount of *E*. *coli O157:H7* on spinach leaves and green pepper slices through around 1–4 logs in the initial research. The initial decline persisted at 4°C, but there was significant regrowth on 25°C. When two or three phages were coupled in the retrial, the prevalence of *E*. *coli* was reduced to extremely low levels in both UHT and unpasteurized milk. In contrast to samples treated with the two‐phage cocktail, where it started to develop again, the *E*. *coli* strain continued to diminish in all samples treated with the three‐phage preparation during storage at both 4 and 25°C. Despite the fact that the fundamental details are not completely tacit, the three‐phage cocktail probably managed resistance better than a two‐phage cocktail (Tomat et al., 2014). In the past, multi‐phage cocktails have been proven to be more effective. The underlying causes of this phenomenon have not been thoroughly investigated. This idea is quite similar to the multi‐hurdle method, which suggests combining various antibacterial tactics to prevent the emergence of bacterial resistance (Snyder et al., 2016).

### 5.4 Bacteriophage to control *Shigella* spp. contamination

Currently, the FDA has only authorized one food safety phage preparation that exclusively targets *Shigella* spp. It was awarded the GRAS certification in 2017 (GRN 672) for this five‐phage cocktail after it remained exposed that the heights of *Shigella* were decreased through around 1 log in a variety of foods, including lettuce, yoghurt, smoked salmon, deli corned beef, melons and chicken breast meat. In a different study, the same combination of *Shigella* specific bacteriophages was used to assess the efficacy of giving phages against pharmaceuticals to mice exposed to a *Shigella sonnei* strain (Soffer et al., 2017). The results of the above research showed that although the Shigella‐specific phages concoction was similarly efficient as just a basic antimicrobial at lowering the amount of bacteria in mice, antimicrobial therapy substantially altered the uniqueness of the cursor bowel society, so although viral diagnosis was doing not. Retroviral management thus was much fairly mild consequence just on mice's regular gut bacteria than antibiotic therapy was doing. The researcher's observations show that the phage had no negative effects on the mice's weight, morbidity, mortality, or any other physiological characteristics. Both the mice's blood and urine contained the same elements as before (Mai et al., 2015).

### 5.5 Bacteriophage to control *Campylobacter* contamination

Prior to the discovery of antibiotics, efforts were made to treat patients using bacteriophages. Bacteriophages have two life cycles, lysogenic and lytic, which may be used in therapeutics. Bacteriophages are characterized by their specificity, which allows them to selectively work against certain bacteria while having no negative effects on the surrounding flora, which is crucial for the advancement of human health. On the other hand, this uniqueness causes certain issues with phage therapy's immunity problem and also necessitates very specialized techniques (Bragg et al., 2014; Hashempour‐Baltork et al., 2019; Lin et al., 2017). The potential to decrease *Campylobacter* adulteration of numerous foods has been investigated for some of the *Campylobacter* bacteriophages that have been inaccessible from chickens, counting their feces and the surface and internal tissues of their livers (Hammerl et al., 2014). For instance, Hammerl and colleagues discovered a significant reduction (3 logs) in Campylobacter fecal levels when two phages were delivered consecutively to 20‐day‐old chicks (a Group III phage, then a Group II phage). It's noteworthy to note that the Group III phage was unsuccessful when given unaided or in combination through additional Group III phage, signifying that a mix of Group II and III phages was obligatory for maximal efficiency. *Campylobacter*‐specific phages have previously been isolated from a small number of Campylobacter isolates, with many investigations using the single isolate of *C*. *jejuni* NCTC 12662 as the host strain. The discovery of several Group III phages, the majority of which target the capsular polysaccharide, a specific receptor, was made possible by that one strain. On the other hand, Group II phages that enter through the flagella are often seen on *C*. *jejuni* RM1221 (Sørensen et al., 2015).

## 6 BACTERIOPHAGES AT THE POST‐HARVEST STAGE OF FOOD PRODUCTION

Depending on the amount of preservatives used, the nutrient‐rich conditions found in food may promote the survival and emergence of several bacterial illnesses. According to the research, using certain phages can help prevent the spread of a number of hazardous diseases. In order to manage infections in postharvest food products, several have outlined intervention procedures that use phages as illustrated in Table 2.

**TABLE 2:** Studies investigating the efficacy of bacteriophages applied to produce safe food.

Product	Bacterial species	Phages	Results	References
Cantalouqe	*Salmonella enteritidis* S3, *javianna* S200 and S203 (10^5^ CFU/mL) Salmonella Newport S195 (10^5^ CFU/mL)	F3, F6, Felix01, HER20, SE13 (2500)	No discernible decline in the pathogen was seen, only a log drop of around 2–3 CFU/cm^2^	Wong et al. (2020)
Cut Spinach	*Listeria monocytogenes* (4.5 Log CFU/cm^2^)	ListShield (2)	After 14 days, a log decrease of 1.51–2.51 CFU/cm^2^ was noted	Boyacioglu et al. (2016)
Lettuce	*Salmonella typhimurium* (10^7^ CFU/mL)	LPST10 (1, 10, 100)	After five hours, log decreases of 1.7 and 2.7 CFU/cm^2^ were noticed	Huang et al. (2018)
Romaine lettuce	*Salmonella* (10^5^ CFU/mL)	SalmoFresh (1000)	2.43 CFU/g of log decrease was seen. 2.16 CFU/g of log decrease was seen	Zhang et al. (2019)
Alfalfa seed and sprout	*Escherichia coli* (10^4^ CFU/mL)	EcoM‐HG2, EcoM‐HG7, EcoM‐HG8 (2.5 × 10^6^)	After 5 days, a log decrease of 1.5 CFU/g was noticed	Lone et al. (2016)
Cabbage	*Salmonella enteriditis* (10^4^ CFU/mL)	PA13076, PC2184 (104)	After 5 h, a log decrease of 3.0–3.86 CFU/sample was noticed	Bao et al. (2015)

### 6.1 Meat

As part of ongoing research on meat systems, researchers done a thorough inspection of the Intralytix phage cocktail EcoShield PXTM with a focus on *E*. *coli* that generates Shiga toxin. In eight various dietary commodities, comprising meat chuck roast, beef mince, chicken breast, cooked chicken, salmon, cheese, cantaloupe, and mustard greens, they discovered that such microbes were effective in reducing pathogen levels (at 3.0 log CFU/g). 97% of the foods were analyzed, and the reductions in *E*. *coli* O157:H7 were substantial (*p* .05) once the phages remained administered at 5106 and 1107 PFU/g. When usual levels of *E*. *coli* (1–10 CFU/10 g) were found in beef parts that were offered for sale, pathogen counts were reduced by 80% (Vikram et al., 2020).

In another study, scientist evaluated the efficacy of four Salmonella phages active against the *serovars Enteritidis*, *Typhimurium*, *Paratyphi A*, *San Diego*, and *Typhi* in chicken breast meat. Phage challenge studies at 4°C produced inconsistent CFU reduction values in Salmonella counts after cold storage of the meat (*p* .05), but they consistently demonstrated the phage's exceptional antibacterial action (Kim et al., 2020). A similar study conducted at 8°C, demonstrated that the value of employing a five‐phage cocktail to manage *Salmonell*a on samples of chicken breast meat by reducing *S*. *enteritidis* and *S*. *typhimurium* on the meat and by observing a statistically significant reduction (*p* .05) of viable counts by 1.41 and 1.86 log CFU/piece, respectively. These findings are in contrast to those obtained using the commercially available SalmoFresh™ (manufactured by Intralytix Inc.), which reduced the levels of Salmonella in infected chicken breasts by up to 1.5 logs (Duc et al., 2020). Researchers conducted research on beef meat to evaluate the effectiveness of a phage cocktail against two Shiga‐toxigenic *E*. *coli* strains and the clinically important Entero‐pathogenic strain. They also examined the results of similar tasks carried out in sterile milk and broth. However, they did note that while trials were effective when conducted at 24 and 37°C, they were less effective at 4°C (Tomat et al., 2018). The commercial product EcoShield PX™ from Intralytix Inc. produced better results, lowering *E. coli* O157:H7 levels on various food products by as much as 97% [18]. In a different investigation, researchers investigated the efficacy of a two‐phage cocktail against *C*. *jejuni* in chicken meat at 5°C. After doing their research, they found that the phage preparation could lower the quantity of *C*. *jejuni* on the infected chicken skin by 0.73 logs (from a starting concentration of 10^4^ CFU/mL). Despite the fact that their tests served as enough proof of concept, they came to the conclusion that a complete comprehension of phage‐host interactions was necessary. Understanding the interactions that must take place between phages and their host during refrigeration is essential for *C*. *jejuni* biocontrol approaches (Zampara et al., 2017).

### 6.2 Fruit and vegetable foods

According to researchers the anti‐Salmonella phage LSE7621's have ability for biocontrol of the pathogen on lettuce, *Salmonella* levels were reduced by 0.86 log10 CFU/mL at a MOI of 100 and by 1.02 log10 CFU/mL at a MOI of 1 after six hours. *Salmonella* counts decreased by 3.55 log10 CFU/mL (MOI = 100) and 1.86 log10 CFU/mL (MOI = 1) after four hours, respectively, according to comparable challenge studies employing tofu (coagulated soy milk; Liu et al., 2020). In a similar study conducted by scientist, a five‐component phage cocktail was used to control seven *S*. *enterica* strains from four different *Serovars enteritidis*, Newport, Javiana, and Thompson—were treated using a five‐part phage cocktail following inoculation into romaine lettuce leaves and cantaloupe. The food samples received the phage combination for 24 h before becoming bacterially infected. Even though the phages appeared to have potential for *Salmonella* biocontrol, the data demonstrated that efficiency varied greatly depending on the target *Salmonella* strain (Wong et al., 2020).

### 6.3 Processed foods

There has been significant advancement in the creation of novel, FDA‐approved phage therapies for a variety of critical disorders. These include *E*. *coli O157:H7*, *Shigella* spp., *Salmonella* spp., and *Listeria monocytogenes*. More evidence in favor of using phages for food safety may be found in the following list of recent studies. Scientists discovered an anti‐*Listeria SH3‐3* phage in a food processing plant and assessed the efficiency of biocontrol against *L*. *monocytogenes* in both salmon and orange juice (Zhou et al., 2020). Researchers demonstrated the anti‐*Salmonella* Enteritidis phage SE07's biocontrol ability in a variety of retail products, such as fruit juice, fresh eggs, beef, and poultry. They also found that fruit juice and fresh eggs had a 2‐log decrease in the germs following a 48‐h test at 4°C. All of the aforementioned research strongly supports the continuous creation of phage products with a wide host range in light of the different potential applications (Thung et al., 2017).

#### 6.3.1 Strength and limitations of phage as antimicrobial

Additionally, since diverse bacterial strains typically inhabit illnesses, this specificity presents a challenge. Even though a small number of trials verified the safety of taking phage orally, the crucial concern is proper phage translocation throughout the intestinal epithelium. Studies have clearly shown that this translocation can benefit the body by controlling the immune response to native microbial antigens by preventing the development of tumor necrosis factor, interleukin‐2, and interferon gamma. Nevertheless, other research did not find an appreciable rise in cytokine levels following phage therapy. Despite the paucity of information about phage treatment, studies have shown that it has far less side effects than conventional antibiotics, in addition to reducing the gut pathogenic flora. Finding the phages with the best infectivity against the target pathogen was made possible by regional specificity. This can be more beneficial when searching for phages for bacteria that are resistant to antibiotics, particularly in hospitals. Moreover, phages include enzymes like extracellular polymeric components depolymerize that may break down bacterial biofilms and extracellular polymeric materials, while antibiotics are unable to cure infections caused by bacteria that form biofilms.

## 7 CONCLUSION

Fresh fruit and vegetable contamination remains a major issue despite recent improvements in food safety procedures. Pathogenic bacteria and rotting bacteria can both lower product quality and increase food waste, respectively. According to recent research in the field of food microbiology, bacteriophages are particularly effective at preventing the formation of dangerous bacteria on fresh vegetables. Bacteriophage usage can be beneficial at several stages of the food production chain. Despite advancements in food safety, foodborne infections remain an issue, especially for those with weakened immune systems, such kids, the elderly, and expectant mothers. Bacteriophage biocontrol is a viable supplementary tool in a multi‐pronged strategy to stop the spread of foodborne infections. This strategy shows the most promise once food mainframes try toward retain the normal, then frequently favorable, bacterial community of nutriments then toward solitary eliminate germs that might reason infection trendy people.

## AUTHOR CONTRIBUTIONS

**Ali Imran:** Writing – original draft (equal). **Umber Shehzadi:** Writing – original draft (equal). **Fakhar Islam:** Writing – original draft (equal). **Muhammad Afzaal:** Formal analysis (equal). **Rehman Ali:** Writing – review and editing (equal). **Yuosra Amer Ali:** Visualization (equal). **Anamika Chauhan:** Validation (equal). **Sunanda Biswas:** Visualization (equal). **Sadaf Khurshid:** Data curation (equal). **Ifrah Usman:** Software (equal). **Ghulam Hussain:** Software (equal). **Syeda Mahvish Zahra:** Investigation (equal). **Mohd Asif Shah:** Data curation (equal). **Adil Rasool:** Validation (equal).

## CONFLICT OF INTEREST STATEMENT

The authors declare no conflict of interest.

## ETHICAL APPROVAL

The study does not involve any human or animal testing.

## CONSENT TO PARTICIPATE

All the co‐authors are willing to participate in this manuscript.

## CONSENT FOR PUBLICATION

All authors are willing for publication of this manuscript.

## Data Availability

The data that support the findings of this study are available from the corresponding author, upon request.
