# Correction to bacteriophages and food safety: An updated overview

**DOI:** 10.1002/fsn3.3649

**Published:** 2023-09-04

Imran, A., Shehzadi, U., Islam, F., Afzaal, M., Ali, R., Ali, Y. A., Chauhan, A., Biswas, S., Khurshid, S., Usman, I., Hussain, G., Zahra, S. M., Shah, M. A., & Rasool, A. (2023). Bacteriophages and food safety: An updated overview. *Food Science and Nutrition*, *11*, 3621–3630. https://doi.org/10.1002/fsn3.3360

The following affiliations were erroneously missing for Mohd Asif Shah: School of Business, Woxsen University, Kamkole, Sadasivpet, Hyderabad, Telangana, India; Division of Research and Development, Lovely Professional University, Phagwara, Punjab, India; and School of Engineering and Technology, Sharda University, Greater Noida, India.

The corrected affiliations appear here:

Ali Imran^1^ | Umber Shehzadi^1^ | Fakhar Islam^1,2^ | Muhammad Afzaal^1^ | Rehman Ali^1^ | Yuosra Amer Ali^3^ | Anamika Chauhan^4,5^ | Sunanda Biswas^6^ | Sadaf Khurshid^1^ | Ifrah Usman^1^ | Ghulam Hussain^7^ | Syeda Mahvish Zahra^8,9^ | Mohd Asif Shah^10,11,12^ | Adil Rasool^13^

^1^Department of Food Sciences, Government College University, Faisalabad, Pakistan

^2^Department of Clinical Nutrition, NUR International University, Lahore, Pakistan

^3^Department of Food Sciences, College of Agriculture and Forestry, University of Mosul, Mosul, Iraq

^4^Department of Home Science, Chaman Lal Mahavidyalaya Landhora, Haridwar, India

^5^Sri Dev Suman University, Tehri, India

^6^Department of Food & Nutrition, Acharya Prafulla Chandra College, Kolkata, India

^7^Neurochemicalbiology and Genetics Laboratory (NGL), Department of Physiology, Faculty of Life Sciences, Government College University, Faisalabad, Pakistan

^8^Department of Environmental Design, Health and Nutritional Sciences, Allama Iqbal Open University, Islamabad, Pakistan

^9^Institute of Food Science and Nutrition, University of Sargodha, Sargodha, Pakistan

^10^University Centre for Research & Development, University School of Business, Chandigarh University, Gharuan, Mohali, Punjab, India

^11^School of Business, Woxsen University, Kamkole, Sadasivpet, Hyderabad, Telangana, India

^12^Division of Research and Development, Lovely Professional University, Phagwara, Punjab, India

^13^Department of Management, Bakhtar University, Kabul, Afghanistan

